# Using Mendelian randomization study to assess the renal effects of antihypertensive drugs

**DOI:** 10.1186/s12916-021-01951-4

**Published:** 2021-03-26

**Authors:** Jie V. Zhao, C. Mary Schooling

**Affiliations:** 1 School of Public Health, Li Ka Shing Faculty of Medicine, The University of Hong Kong, 1/F, Patrick Manson Building, 7 Sassoon Road, Hong Kong, China; 2 School of Public Health and Health Policy, City University of New York, New York, NY USA

**Keywords:** Antihypertensives, Kidney function, Mendelian randomization

## Abstract

**Background:**

Angiotensin-converting enzyme (ACE) inhibitors and/or in combination with calcium channel blockers (CCBs) are generally recommended as the first-line antihypertensive therapy for people with hypertension and kidney dysfunction. Evidence from large randomized controlled trials comprehensively comparing renal effects of different classes of antihypertensive drugs is lacking.

**Methods:**

We used a Mendelian randomization study to obtain unconfounded associations of genetic proxies for antihypertensives with kidney function. Specifically, we used published genetic variants in genes regulating target proteins of these drugs and then applied to a meta-analysis of the largest available genome-wide association studies of kidney function (estimated glomerular filtration rate (eGFR), urine albumin-to-creatinine ratio (UACR), and albuminuria). Inverse variance weighting was used as the main analysis and to combine estimates from different sources.

**Results:**

Genetically predicted ACE inhibition was associated with higher eGFR (effect size 0.06, 95% confidence interval (CI) 0.008, 0.11), while genetic proxies for beta-blockers were associated with lower eGFR (− 0.02, 95% CI − 0.04, − 0.004) when meta-analyzing the UK Biobank and CKDGen. Genetic proxies for CCBs were associated with lower UACR (− 0.15, 95% CI − 0.28, − 0.02) and lower risk of albuminuria (odds ratio 0.58, 95% CI 0.37, 0.90) in CKDGen. The associations were robust to using different analysis methods and different genetic instruments.

**Conclusions:**

Our findings suggest the reno-protective associations of genetically proxied ACE inhibitors and CCBs, while genetic proxies for beta-blockers may be related to lower eGFR. Understanding the underlying mechanisms would be valuable, with implications for drug development and repositioning of treatments for kidney disease.

**Supplementary Information:**

The online version contains supplementary material available at 10.1186/s12916-021-01951-4.

## Background

Hypertension is a leading contributor to global years of life lost because of its role in cardiovascular disease [1]. Hypertension is also a key risk factor for impaired kidney function, which might also be affected by kidney function [2]. Different classes of antihypertensive drugs, acting via different targets, may have different renal effects. Most guidelines recommend the use of angiotensin-converting enzyme (ACE) inhibitors as the first-line antihypertensive therapy for the treatment of hypertension in patients with chronic kidney disease (CKD) [3]. The European Society of Cardiology (ESC)/European Society of Hypertension (ESH) guidelines also advocate the combination of an ACE inhibitor with a calcium channel blocker (CCB) as the first-line therapy in patients with proteinuria [4]. In randomized controlled trials (RCTs), treatment with ACE inhibitors can slow down the decline in glomerular filtration rate (GFR) [5] and the progression to end-stage renal failure [6], independent of the reduction in blood pressure [5]. Network meta-analysis of RCTs comparing different classes of antihypertensives suggests that the ACE inhibitor-CCB combination therapy is the most efficacious therapy in reducing albuminuria in patients with diabetes and microalbuminuric kidney disease [7], while whether the findings can be generalized to people without these comorbidities is unclear. Given the limited number of often small-scale RCTs, current evidence may not be sufficient to support the use of ACE inhibitors as the first-line antihypertensive drug [8]. Evidence from large RCTs comprehensively comparing the efficacy of different classes of antihypertensives in kidney function is lacking.

In these circumstances, Mendelian randomization (MR) which exploits drug target-related genetic variants to mimic drug effects provides an alternative approach. As genetic variants are randomly allocated at conception and unlikely affected by socioeconomic position or other confounders, the study design minimizes residual confounding and has been successfully applied previously to assess the cardiovascular effects of several drugs such as antihypertensives and lipid-lowering medications [9, 10]. Here, we used published genetic variants, likely corresponding to the effects of antihypertensives [11, 12], to assess the association of genetic proxies for ACE inhibitors and CCBs with kidney function. We also assessed the associations of genetic proxies for other antihypertensives with kidney function for completeness.

## Methods

### Study design

We used an MR study to obtain unconfounded associations of genetic proxies for antihypertensives (ACE inhibitors, angiotensin II receptor blockers (ARBs), CCBs, alpha-adrenoceptor blockers, adrenergic neuron blocking drugs, beta-adrenoceptor blockers (BBs), centrally acting antihypertensive drugs, loop diuretics, potassium-sparing diuretics (PSDs) and aldosterone antagonists, renin inhibitors, thiazides and related diuretics, and vasodilator antihypertensives) with kidney function. Specifically, we used published genetic variants in genes regulating the drug target proteins [11, 12], as proxies for different classes of antihypertensive drugs, and then assessed the associations of these genetic variants with estimated glomerular filtration rate (eGFR) in the UK Biobank and CKDGen Consortium (“CKDGen”), with urine albumin-to-creatinine ratio (UACR) and albuminuria in CKDGen.

### Study population

The UK Biobank is a large, ongoing, prospective cohort study, with a median follow-up time of 11.1 years [13]. It recruited 502,713 people (intended to be aged 40–69 years, mean age 56.5 years, 45.6% men) from 2006 to 2010 in England, Scotland, and Wales, 94% of self-reported European ancestry. CKDGen is a large, trans-ancestry genome-wide association study (GWAS) meta-analysis comprising 121 GWAS summary statistics for eGFR in 765,348 people, 567,460 of them of European ancestry, with a median age of 54 years, 50% men [14], and 54 GWAS summary statistics for UACR in 564,257 people, 547,361 of European ancestry [15]. GWAS summary statistics for albuminuria were from the meta-analysis of GWAS (51,861 cases, 297,093 controls), with adjustment for age, sex, study-specific covariates, and genetic principal components [15]. To avoid population stratification, we only considered people of white British ancestry in the UK Biobank and of European ancestry in CKDGen (Additional file 1: Table S1).

### Exposure

We obtained genetic proxies (single nucleotide polymorphisms (SNPs)) for ACE inhibitors, ARBs, CCBs, alpha-adrenoceptor blockers, adrenergic neuron blocking drugs, BBs, centrally acting antihypertensive drugs, loop diuretics, PSDs and aldosterone antagonists, renin inhibitors, thiazides and related diuretics, and vasodilator antihypertensives from published sources [11, 12]. Specifically, these published studies give the genetic variants regulating the expression of the relevant drug target genes and selected genetic variants related to systolic blood pressure (SBP) in different studies (UK Biobank summary statistics released in 2017 [11] or meta-analysis of the UK Biobank and the International Consortium of Blood Pressure GWAS [12]). For validity, we further checked and selected SNPs also related to SBP in the latest UK Biobank summary statistics of people of European ancestry in Pan UKBB. The strength of each genetic variant was assessed from the *F*-statistic obtained using an established approximation as previously [16]; only genetic variants with *F*-statistic > 10 were used. Details concerning these genetic variants are in Additional file 1: Table S2. ACE inhibitors, ARBs, and renin inhibitors have only one proxy, given the insufficient power [17], as previously [16] we did not include them for the categorical outcome, i.e., albuminuria. To increase the number of genetic variants available, in sensitivity analysis, we included correlated genetic variants with *r*^2^ < 0.8, taking into account their correlations obtained from 1000 Genomes European panel using “ld_matrix.” We used Steiger filtering which enables inference of the causal direction, by calculating and comparing the variance explained by the genetic instrument in kidney function and in blood pressure [18]. To assess potential pleiotropy, we searched a curated genotype to phenotype cross-reference, Phenoscanner (http://www.phenoscanner.medschl.cam.ac.uk) and summary statistics for creatinine from the UK Biobank, to identify whether the genetic variants (or their proxies (*r*^2^ > 0.8)) were associated with other risk factors for kidney function or disease at genome-wide significance. We conducted the analysis by drug class and also by drug target within each drug class and combined the estimates for all drug classes to obtain the overall association of genetic proxies for antihypertensive drugs with kidney function. We also assessed the association of genetic predictors of SBP, identified from a GWAS of SBP without adjustment for antihypertensive medication or BMI (Additional file 1: Table S1) [16, 19], with kidney function.

From the studies based on a GWAS meta-analysis of the UK Biobank and the International Consortium of Blood Pressure (ICBP) [12, 16], we also checked the SNP selection using the recent GWAS meta-analysis of the UK Biobank and ICBP [20], to identify any additional genetic instruments. For ease of comparison, we retained variants with *r*^2^ < 0.01, obtained using “ld_clump,” for both sets of genetic instruments selection. Details of these genetic instruments are in Additional file 1: Table S2.

### Outcomes

The outcomes include eGFR, albuminuria, and UACR. Genetic associations with eGFR were obtained from the UK Biobank individual-level data (application #42468) and from CKDGen summary statistics. Genetic associations with UACR and albuminuria were obtained from CKDGen summary statistics [15]. eGFR was calculated based on the CKD-EPI formula using serum creatinine [21]. UACR was calculated using urine albumin divided by creatinine. Albuminuria was defined as UACR > 30 mg/g [22].

### Statistical analysis

MR estimates were based on the SNP-specific Wald estimates, i.e., the genetic association with kidney function divided by the genetic association with the genetic proxies for antihypertensives. The MR estimates are presented in effect size of SBP, obtained from the latest Pan UK Biobank summary statistics (https://pan.ukbb.broadinstitute.org/downloads/index.html), of European ancestry, adjusted for age, age^2^, sex, interaction of sex with age, age^2^, and 20 principal components.

We used linear regression to assess the association of each genetic variant with log-transformed eGFR, controlling for age, sex, 20 principal components, and assay array. In CKDGen, the meta-analysis used linear regression for genetic associations with log-transformed eGFR and UACR, controlling for age, sex, genetic principal components, relatedness, and other study-specific characteristics as appropriate [14]. We meta-analyzed the SNP-specific Wald estimates using inverse variance weighting (IVW) with multiplicative random effects, as necessary. To maximize power, MR estimates for eGFR from different studies were meta-analyzed together as the main analysis.

In the sensitivity analysis, we used different methods to control for pleiotropy, including a weighted median and Mendelian randomization pleiotropy residual sum and outlier (MR-PRESSO), where applicable. The weighted median estimate is robust to invalid instruments and able to provide consistent estimation even when up to 50% of the weight is from invalid SNPs [23]. MR-PRESSO is able to identify outliers with potential horizontal pleiotropy among multiple genetic variants and provide a corrected estimate after removing these outliers [24].

All statistical analyses were conducted using R version 4.0.1 (R Foundation for Statistical Computing, Vienna, Austria) and the R package “MendelianRandomization.”

## Results

**Fig. 1:**
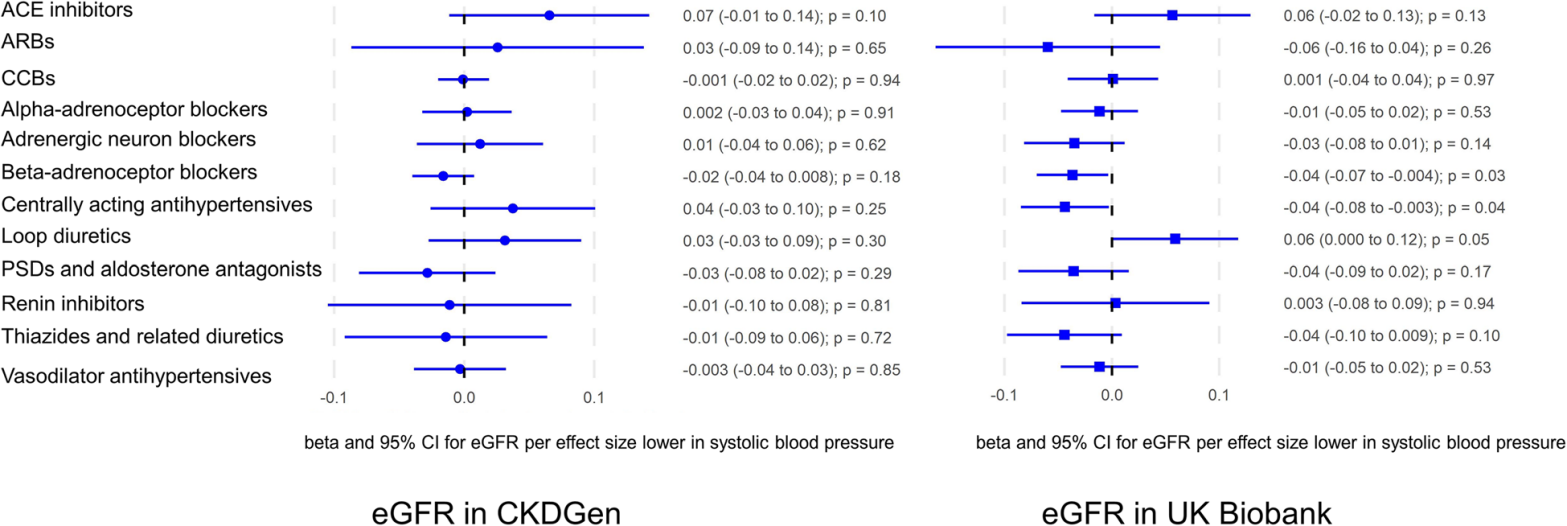
Associations of genetic proxies for antihypertensive drugs with eGFR in the UK Biobank and CKDGen

Genetically predicted ACE inhibition was associated with higher eGFR in the meta-analysis of the UK Biobank and CKDGen using uncorrelated variant and including a correlated genetic variant (rs4311 in *ACE*) (Table 1 and Additional file 1: Table S3). Genetic proxies for loop diuretics and BBs were associated with higher and lower eGFR respectively (Table 1). The associations were generally consistent in the UK Biobank and CKDGen (Fig. 1). Genetic proxies for CCBs were associated with lower UACR and lower risk of albuminuria in CKDGen (Tables 2 and 3). The estimates were similar after incorporating correlated genetic variants (Additional file 1: Tables S3-S5). Steiger filtering indicated directionality from genetic proxies for antihypertensives to kidney function. Five genetic variants (4 for CCBs and 1 for vasodilator antihypertensives) had potentially pleiotropic associations (Additional file 1: Table S6). Sensitivity analysis excluding them did not change the conclusion (Additional file 1: Tables S7 and S8). The estimates were similar using a weighted median and MR PRESSO (Additional file 1: Tables S9 and S10).

**Table 1:** Associations of genetic proxies for antihypertensive drugs with eGFR in a meta-analysis of the UK Biobank and CKDGen

Class	#SNPs	Beta	95% CI	*p*
ACE inhibitors	1	0.06	0.008, 0.11	0.02
ARBs	1	−0.02	− 0.10, 0.06	0.65
CCBs	9	−0.0001	−0.03, 0.03	0.99
Alpha-adrenoceptor blockers	6	−0.004	−0.03, 0.02	0.73
Adrenergic neuron blockers	3	−0.01	−0.06, 0.03	0.61
Beta-adrenoceptor blockers	7	−0.02	−0.04, − 0.004	0.02
Centrally acting antihypertensives	4	−0.01	−0.09, 0.07	0.86
Loop diuretics	2	0.05	0.004, 0.09	0.03
PSDs and aldosterone antagonists	4	−0.03	−0.07, 0.005	0.09
Renin inhibitors	1	−0.003	−0.07, 0.06	0.92
Thiazides and related diuretics	4	−0.03	−0.08, 0.01	0.12
Vasodilator antihypertensives	8	−0.01	−0.03, 0.02	0.57

Beta is the beta-coefficient with eGFR per effect size (standard deviation) of systolic blood pressure

*ACE*, angiotensin-converting enzyme; *ARB*, angiotensin II receptor blocker; *CCB*, calcium channel blocker; *eGFR*, estimated glomerular filtration rate; *PSD*, potassium-sparing diuretic

**Table 2:** Associations of genetic proxies for antihypertensive drugs with UACR in CKDGen

Class	#SNPs	Beta	95% CI	*p*
ACE inhibitors	1	0.07	−0.37, 0.51	0.76
ARBs	1	−0.06	−0.70, 0.58	0.86
CCBs	9	−0.15	−0.28, − 0.02	0.03
Alpha-adrenoceptor blockers	6	−0.10	−0.25, 0.05	0.17
Adrenergic neuron blockers	3	−0.12	−0.41, 0.16	0.39
Beta-adrenoceptor blockers	7	−0.04	−0.17, 0.10	0.58
Centrally acting antihypertensives	4	−0.24	−0.49, 0.01	0.06
Loop diuretics	2	0.17	−0.17, 0.52	0.33
PSDs and aldosterone antagonists	4	−0.14	−0.56, 0.29	0.53
Renin inhibitors	1	−0.27	−0.80, 0.27	0.33
Thiazides and related diuretics	4	−0.10	−0.52, 0.32	0.63
Vasodilator antihypertensives	8	−0.07	−0.23, 0.09	0.39

*ACE*, angiotensin-converting enzyme; *ARB*, angiotensin II receptor blocker; *CCB*, calcium channel blocker; *PSD*, potassium-sparing diuretic; *UACR*, urine albumin-to-creatinine ratio

**Table 3:** Associations of genetic proxies for antihypertensive drugs with albuminuria

Class	#SNPs	Odds ratio	95% CI	*p*
CCBs	8	0.58	0.37, 0.90	0.01
Alpha-adrenoceptor blockers	6	0.81	0.41, 1.60	0.54
Adrenergic neuron blockers	3	1.45	0.56, 3.75	0.44
Beta-adrenoceptor blockers	7	0.71	0.35, 1.46	0.35
Centrally acting antihypertensives	4	0.54	0.21, 1.39	0.20
Loop diuretics	2	1.39	0.43, 4.52	0.58
PSDs and aldosterone antagonists	3	1.17	0.41, 3.37	0.77
Thiazides and related diuretics	4	0.81	0.31, 2.11	0.66
Vasodilator antihypertensives	8	1.04	0.51, 2.14	0.92

*CCB*, calcium channel blocker; *PSD*, potassium-sparing diuretic

The associations of genetically predicted ACE inhibition and BBs with eGFR, as well as the associations of genetic proxies for CCBs with UACR and albuminuria, were replicated using another set of genetic variants based on a meta-analysis of the UK Biobank and ICBP [12, 16, 20] (Additional file 1: Figure S1). These genetic proxies for CCBs also had a positive association with eGFR (Additional file 1: Figure S1).

Genetic proxies for antihypertensive drugs overall had no association with eGFR (Additional file 1: Figure S2). Genetically predicted SBP was associated with lower eGFR using MR PRESSO (Additional file 1: Figure S2). Genetic proxies for antihypertensive drugs overall were associated with lower UACR and lower risk of albuminuria (Additional file 1: Figures S3 and S4), while genetically predicted SBP was associated with higher UACR and higher risk of albuminuria (Additional file 1: Figures S3 and S4).

## Discussion

Using MR study to minimize residual confounding, this study suggests that genetically predicted ACE inhibition and genetic proxies for CCBs are beneficial for kidney function. The findings are consistent with the current guidelines concerning the use of antihypertensives in CKD and evidence from RCTs [5–7]. Consistent with previous concerns about BBs [25, 26], this study also suggests that genetic proxies for BBs may relate to lower eGFR, although the clinical significance of the small effect size is unclear. The null association of genetic proxies for antihypertensives overall with eGFR might be due to the varying associations in different classes of antihypertensives.

Our findings suggest that genetic proxies for ACE inhibitors and CCB have more beneficial associations with kidney function than genetic proxies for other classes of antihypertensive drugs. Kidney dysfunction relates to a higher risk of cerebrovascular disease [27]; consistently, genetic proxies for CCBs are associated with a lower risk of cerebrovascular disease [16]. The mechanisms underlying the beneficial associations remain to be explored. Understanding the mechanisms is also helpful when there are varying associations of proxies in different genes, such as in genetic proxies for CCBs and eGFR. In the RCT of ramipril (an ACE inhibitor), its effect on slowing the decline of GFR was not fully explained by lowering blood pressure [5]. The beneficial associations for ACE inhibitors and CCBs are also possibly through mechanisms beyond lowering blood pressure, such as via the regulation of the renin-angiotensin-aldosterone system (RAAS). RAAS may affect kidney function by several mechanisms, such as via pro-inflammation, endothelial dysfunction, and increasing glomerular capillary pressure [28]. As such, ACE inhibitors may have a reno-protective role by inhibition of RAAS. However, an RCT of dual blockade of RAAS with ACE inhibitors and ARB did not exhibit more benefits for kidney function than monotherapy [29], raising the possibility that other pathways might exist. Notably, CKD has an apparent sex disparity, and testosterone has been recognized as a causal factor explaining, or partly explaining, unfavorable kidney function in men [30, 31]. ACE inhibitors and CCB may also play a role by modulating sex hormones. ACE inhibitors lower free testosterone in men and increase sex hormone-binding globulin in women [32]. CCBs are also known to have an anti-reproductive effect in men [33] and to lower testosterone in animal experiments [34], but the effect remains to be examined in humans.

Despite using MR to minimize confounding and consistency with RCTs [5–7], this study has several limitations. First, MR relies on three assumptions, i.e., the genetic instruments relate to the exposure, are not related to the potential confounders, and the association of the genetic instruments with the outcome is exclusively through the exposure [35]. To satisfy these assumptions, we used published SNPs related to the expression of genes regulating the relevant antihypertensive target proteins. Moreover, the beneficial associations of genetically proxied ACE inhibition with eGFR and genetic proxies for CCBs with UACR and albuminuria were consistent using different genetic instruments derived from different studies [11, 12]. Second, weak instruments might bias toward the null; however, the genetic variants we used had *F*-statistics > 10. MR estimates are less precise than conventional observational studies, although less prone to confounding, because the genetic variants can only explain a small proportion of the variance in exposure. As such, as previously [16], we did not conduct an analysis for antihypertensive drugs with only one genetic proxy for the categorical outcome [16], albuminuria. Although we used by far the largest study for kidney function, i.e., a meta-analysis of the UK Biobank and CKDGen Consortium, the null associations should be interpreted with caution, and we cannot exclude the role of some antihypertensive drugs. Third, most participants in the UK Biobank do not have kidney disease [36], so the MR estimates from the UK Biobank might be more applicable to the general population than to patients with kidney disease. However, the directions of associations should be consistent across populations. For example, the benefits of ACE inhibitors in kidney function shown in this MR study were also evident in an RCT targeting patients with proteinuric nephropathy [5]. Fourth, when using the UK Biobank, the estimates might be biased because the genetic predictors for antihypertensives and genetic associations with kidney function were from the same study [37]. However, the associations using CKDGen were similar, whose participants may not overlap with those in the UK Biobank. Fourth, the associations in Europeans may not apply to other populations, such as Asians. However, causal effects should be consistent across settings, unless the underlying mechanisms and targets vary by setting. Replication in other ancestries will be worthwhile. Fifth, genetic effects might be diluted by compensatory processes or feedback mechanisms [38]. Such compensation would be expected to mitigate genetic effects, biasing toward the null [39], but does not explain the associations for specifically genetically proxied ACE inhibitors, CCBs, and BBs. Sixth, the associations are relatively small, which may not be clinically significant, but might be relevant to population health [40]. Moreover, MR studies assess lifelong effects so the magnitude of effect sizes might not be comparable to short-term effects of taking antihypertensive drugs, so this study is more relevant to assessing the directions of associations than to providing the magnitude of associations. Given the limited evidence from RCTs with generally small sample sizes [5–7], the beneficial associations of genetic proxies for ACE inhibitors and CCBs in comparison with other classes of antihypertensives provide support for the current clinical guidelines on the treatment of hypertension when it occurs with chronic kidney disease. Finally, although men are more vulnerable to kidney dysfunction, we did not conduct sex-specific analysis because we have not identified sex-specific genetic predictors of the effects of antihypertensives.

From the perspective of clinical practice, our findings, together with previous RCTs [5–7], add support to guidelines recommending the use of ACE inhibitors or ACE inhibitor plus CCB in people with hypertension and kidney dysfunction. Our findings also suggest these reno-protective associations are also generalizable to the general population. Genetic proxies for BBs were associated with lower eGFR; however, given the cardiovascular benefit of BBs [12], this concern should not outweigh its benefits, especially for patients with cardiovascular disease and without other renal comorbidities.

## Conclusions

Our findings suggest reno-protective associations of genetically proxied ACE inhibitors and CCBs with kidney function, while genetic proxies for BBs may be related to lower eGFR. Understanding the mechanisms underlying the reno-protective association of genetic proxies for ACE inhibitors and CCBs in the context of the relative merits of different hypertensives in promoting population health would be valuable, with implications for drug development and repositioning in the treatment of kidney disease.

## Supplementary Information

**Additional file 1: Table S1.** Summary of genome-wide association studies included in this study**. Table S2.** Genetic predictors for different classes of antihypertensives. **Table S3.** Associations of genetic proxies for antihypertensive drugs with eGFR in meta-analysis of UK Biobank and CKDGen incorporating correlated genetic variants**. Table S4.** Associations of genetic proxies for antihypertensive drugs with UACR in CKDGen incorporating correlated genetic variants. **Table S5.** Associations of genetic proxies for antihypertensive drugs with albuminuria incorporating correlated genetic variants**. Table S6.** Genetic predictors with potential pleiotropy**. Table S7.** Sensitivity analysis for the association with eGFR excluding potentially pleiotropic genetic predictors. **Table S8.** Sensitivity analysis for the association with UACR and albuminuria excluding potentially pleiotropic genetic predictors. **Table S9.** Sensitivity analysis on the associations of genetic proxies for antihypertensive drugs with eGFR using published genetic variants derived from UK Biobank in Mendelian randomization using different analysis methods**. Table S10.** Sensitivity analysis on the associations of genetic proxies for antihypertensive drugs with albuminuria and UACR using genetic variants derived from UK Biobank in Mendelian randomization using different analysis methods. **Figure S1.** Comparison using different sets of genetic proxies for ACE inhibitors, CCBs and BBs. (a) for eGFR, (b) for UACR, (c) for albuminuria. Black square refers to using genetic variants based on the study of Walker et al. [11] in UK Biobank, grey circle refers to using genetic variants based on the study of Gill et al. [12] and Georgakis and Gill et al. [16] in meta-analysis of UK Biobank and ICBP. **Figure S2.** Associations of genetic proxies for antihypertensive drugs with eGFR by drug target in each drug class, overall antihypertensives and systolic blood pressure. SBP, systolic blood pressure. ACE, Angiotensin-converting enzyme; ARB, Angiotensin II Receptor Blocker; BBs, beta-adrenoceptor blockers; CCBs, calcium channel blockers; PSDs, potassium-sparing diuretics; SBP, systolic blood pressure. **Figure S3.** Associations of genetic proxies for antihypertensive drugs with UACR by drug target in each drug class, overall antihypertensives and systolic blood pressure. ACE, Angiotensin-converting enzyme; ARB, Angiotensin II Receptor Blocker; BBs, beta-adrenoceptor blockers; CCBs, calcium channel blockers; PSDs, potassium-sparing diuretics; SBP, systolic blood pressure. **Figure S4.** Associations of genetic proxies for antihypertensive drugs with albuminuria by drug target in each drug class, overall antihypertensives and systolic blood pressure. BBs, beta-adrenoceptor blockers; CCBs, calcium channel blockers; PSDs, potassium-sparing diuretics; SBP, systolic blood pressure.

## Data Availability

The access of data from the UK Biobank can be obtained by application to the UK Biobank (http://biobank.ctsu.ox.ac.uk/crystal/). The summary statistics can be downloaded from the website https://pan.ukbb.broadinstitute.org/downloads/index.html;https://ckdgen.imbi.uni-freiburg.de/.

## Abbreviations

ACE: Angiotensin-converting enzyme
ARB: Angiotensin II receptor blocker
BMI: Body mass index
CCB: Calcium channel blocker
CKD: Chronic kidney disease
eGFR: Estimated glomerular filtration rate
ESC: European Society of Cardiology
ESH: European Society of Hypertension
GFR: Glomerular filtration rate
GWAS: Genome-wide association study
MR: Mendelian randomization
PSD: Potassium-sparing diuretic
RCT: Randomized controlled trial
SBP: Systolic blood pressure
SNP: Single nucleotide polymorphism
UACR: Urine albumin-to-creatinine ratio
