# Supplementary material for: Using Mendelian randomization study to assess the renal effects of antihypertensive drugs
Source: BMC Med. 2021 Mar 26;19:79. doi: 10.1186/s12916-021-01951-4 (PMC7995783; doi:10.1186/s12916-021-01951-4)
Supplement: Supplementary file 1 — Additional file 1: Table S1. Summary of genome-wide association studies included in this study. Table S2. Genetic predictors for different classes of antihypertensives. Table S3. Associations of genetic proxies for antihypertensive drugs with eGFR in meta-analysis of UK Biobank and CKDGen incorporating correlated genetic variants. Table S4. Associations of genetic proxies for antihypertensive drugs with UACR in CKDGen incorporating correlated genetic variants. Table S5. Associations of genetic proxies for antihypertensive drugs with albuminuria incorporating correlated genetic variants. Table S6. Genetic predictors with potential pleiotropy. Table S7. Sensitivity analysis for the association with eGFR excluding potentially pleiotropic genetic predictors. Table S8. Sensitivity analysis for the association with UACR and albuminuria excluding potentially pleiotropic genetic predictors. Table S9. Sensitivity analysis on the associations of genetic proxies for antihypertensive drugs with eGFR using published genetic variants derived from UK Biobank in Mendelian randomization using different analysis methods. Table S10. Sensitivity analysis on the associations of genetic proxies for antihypertensive drugs with albuminuria and UACR using genetic variants derived from UK Biobank in Mendelian randomization using different analysis methods. Figure S1. Comparison using different sets of genetic proxies for ACE inhibitors, CCBs and BBs. (a) for eGFR, (b) for UACR, (c) for albuminuria. Black square refers to using genetic variants based on the study of Walker et al. [11] in UK Biobank, grey circle refers to using genetic variants based on the study of Gill et al. [12] and Georgakis and Gill et al. [16] in meta-analysis of UK Biobank and ICBP. Figure S2. Associations of genetic proxies for antihypertensive drugs with eGFR by drug target in each drug class, overall antihypertensives and systolic blood pressure. SBP, systolic blood pressure. ACE, Angiotensin-converting enzyme; ARB, [file 12916_2021_1951_MOESM1_ESM.pdf]

Additional File 1

Table S1. Summary of genome-wide association studies included in this study

Sources for exposure and outcome	Information of genome-wide association study (GWAS)			
Exposure	Source and phenotype	Sample size	Controlled for	ancestry
Genetic proxies for antihypertensive drugs	Systolic blood pressure (SBP) (automatic reading) in UK Biobank (Pan-UK Biobank analysis in people of European ancestry)	457,240, 396,663 are of European ancestry	Age, sex, age * sex, age^2, age^2 * sex, the first 10 PCs	European
	SBP in GWAS meta-analysis of International Consortium of Blood Pressure (ICBP) and UK Biobank. For all UKBB participants that were on antihypertensive medication at time of blood pressure measurement 15mmHg were added to the mean observed SBP [20].	1.3 million	age, age^2, sex, body mass index (BMI), principal components, study-specific covariates	European
Genetic predictors for SBP (in sensitivity analysis)	SBP in UK Biobank (MRC-IEU analysis)	436,419	Genotype array, sex and the first 10 PCs (out of 40) supplied by UK Biobank [19]	European
Outcome				
eGFR*	Log transformed eGFR, calculated based on creatinine, in CKDGen Consortium	765,348 people, 567,460 of European ancestry	age, sex, genetic principal components, relatedness and other study-specific characteristics as appropriate [14]	European
UACR	UACR, calculated based on albuminuria and creatinine, in CKDGen Conortium	564,257 people, 547,361 of European ancestry		European
Albuminuria	Albuminuria in GWAS meta-analysis in CKDGen Consortium. Cases were defined as UACR > 30 mg/g, and controls as UACR < 10 mg/g, no other exclusions were applied [15].	51,861 cases, 297,093 controls	age, sex, study-specific covariates and genetic principal components	Trans-ancestry, largely of European

*We also obtained genetic associations with log transformed eGFR calculated based on creatinine, using individual level data in UK Biobank (under application #42468) in 391,981 people of European ancestry, controlling for genotype array, age, sex and the first 20 PCs. It was not listed here because it was only used for this MR study rather than a genome-wide association study of kidney function.

Table S2. Genetic predictors for different classes of antihypertensives

Drug	Genetic instrument*	rsid	Effect allele	gene	AF	beta	se	R^2	F	Correlated
ACE inhibitors	set1	rs4311	T	*ACE*	0.53	-0.01	0.002	2.71E-05	10.7	1
ACE inhibitors	set1	rs4968783	A	*ACE*	0.62	-0.01	0.002	4.04E-05	16.0	0
ARB	set1	rs118123032	T	*AGTR1*	0.03	-0.02	0.007	1.95E-05	7.7	0
Adrenergic neurone blockers	set1	rs12416245	G	*ADRA2A*	0.19	0.01	0.003	3.08E-05	12.2	1
Adrenergic neurone blockers	set1	rs145279507	T	*SLC6A2*	0.02	-0.03	0.010	3.07E-05	12.2	1
Adrenergic neurone blockers	set1	rs62153010	A	*ADRA2B*	0.17	-0.01	0.003	3.62E-05	14.4	1
Alpha-adrenoceptor blockers	set1	rs12416245	G	*ADRA2A*	0.19	0.01	0.003	3.08E-05	12.2	0
Alpha-adrenoceptor blockers	set1	rs13159674	T	*ADRA1B*	0.20	0.01	0.003	2.54E-05	10.1	0
Alpha-adrenoceptor blockers	set1	rs17645325	C	*ADRA1B*	0.13	0.02	0.004	6.90E-05	27.4	0
Alpha-adrenoceptor blockers	set1	rs1899494	A	*ADRA1A*	0.26	0.01	0.003	3.47E-05	13.8	0
Alpha-adrenoceptor blockers	set1	rs4930012	G	*TH*	0.03	0.02	0.007	2.60E-05	10.3	0
Alpha-adrenoceptor blockers	set1	rs71455916	A	*TH*	0.19	0.02	0.003	0.000175	69.6	0
Alpha-adrenoceptor blockers	set1	rs77185818	A	*ADRA1A*	0.12	-0.01	0.004	3.31E-05	13.1	1
Beta-adrenoceptor blockers	set1	rs151591	C	*ADRB1*	0.77	0.01	0.003	5.63E-05	22.3	1
Beta-adrenoceptor blockers	set1	rs17875422	C	*ADRB1*	0.04	-0.03	0.006	4.75E-05	18.9	1
Beta-adrenoceptor blockers	set1	rs180929	T	*ADRB1*	0.63	0.01	0.002	4.87E-05	19.3	1
Beta-adrenoceptor blockers	set1	rs2024183	C	*ADRB1*	0.13	-0.01	0.004	2.85E-05	11.3	1
Beta-adrenoceptor blockers	set1	rs60580948	A	*ADRB2*	0.14	-0.01	0.003	4.55E-05	18.1	1
Beta-adrenoceptor blockers	set1	rs72823013	G	*ADRB1*	0.13	0.01	0.004	2.66E-05	10.6	1
Beta-adrenoceptor blockers	set1	rs77185818	C	*ADRA1A*	0.12	-0.01	0.004	3.31E-05	13.1	1
Beta-adrenoceptor blockers	set1	rs13159674	G	*ADRA1B*	0.20	0.01	0.003	2.54E-05	10.1	0
Beta-adrenoceptor blockers	set1	rs17645325	G	*ADRA1B*	0.13	0.02	0.004	6.90E-05	27.4	0
Beta-adrenoceptor blockers	set1	rs1899494	A	*ADRA1A*	0.26	0.01	0.003	3.47E-05	13.8	0
Beta-adrenoceptor blockers	set1	rs35866749	G	*ADRB3*	0.16	-0.01	0.003	2.76E-05	10.9	0
Beta-adrenoceptor blockers	set1	rs7076938	A	*ADRB1*	0.74	0.02	0.003	0.000185	73.5	0
Beta-adrenoceptor blockers	set1	rs74717224	A	*ADRB1*	0.06	-0.02	0.005	3.40E-05	13.5	0
Beta-adrenoceptor blockers	set1	rs7737361	A	*ADRB2*	0.19	-0.01	0.003	4.85E-05	19.2	0
Beta-adrenoceptor blockers	set2	rs11196549	A	*ADRB1*	0.04	0.04	0.006	1.50E-04	113.7	0
Beta-adrenoceptor blockers	set2	rs1801253	C	*ADRB1*	0.74	0.02	0.003	5.00E-04	366.8	0
Calcium channel blockers	set1	rs10848645	A	*CACNA1C*	0.55	0.01	0.002	4.47E-05	17.7	0

Calcium channel blockers	set1	rs6445583	A	*CACNA1D*	0.75	0.02	0.003	9.21E-05	36.5	0
Calcium channel blockers	set1	rs79020595	C	*CACNA1D*	0.02	-0.04	0.010	4.35E-05	17.3	0
Calcium channel blockers	set1	rs9890200	C	*CACNA1G*	0.37	-0.01	0.002	6.05E-05	24.0	0
Calcium channel blockers	set1	rs117177120	A	*CACNA1H*	0.06	0.02	0.005	5.64E-05	22.4	0
Calcium channel blockers	set1	rs34484573	A	*CACNA2D2*	0.13	-0.02	0.004	0.000124	49.2	0
Calcium channel blockers	set1	rs10764322	G	*CACNB2*	0.31	0.02	0.003	0.000117	46.6	0
Calcium channel blockers	set1	rs7922241	G	*CACNB2*	0.79	0.01	0.003	5.41E-05	21.5	0
Calcium channel blockers	set1	rs12317778	C	*CACNB3*	0.08	-0.02	0.004	7.88E-05	31.3	0
Calcium channel blockers	set1	rs10828906	T	*CACNB2*	0.74	0.01	0.003	4.17E-05	16.5	1
Calcium channel blockers	set1	rs11012761	A	*CACNB2*	0.10	0.01	0.004	2.92E-05	11.6	1
Calcium channel blockers	set1	rs11012864	C	*CACNB2*	0.16	0.02	0.003	8.06E-05	32.0	1
Calcium channel blockers	set1	rs12416030	C	*CACNB2*	0.20	0.01	0.003	4.11E-05	16.3	1
Calcium channel blockers	set1	rs12772058	T	*CACNB2*	0.16	0.01	0.003	2.85E-05	11.3	1
Calcium channel blockers	set1	rs140766681	C	*CACNB2*	0.02	0.03	0.008	3.37E-05	13.4	1
Calcium channel blockers	set1	rs184067968	A	*CACNB2*	0.02	-0.03	0.010	3.00E-05	11.9	1
Calcium channel blockers	set1	rs198535	G	*CACNA1G*	0.42	-0.01	0.002	5.87E-05	23.3	1
Calcium channel blockers	set1	rs2255266	C	*CACNB2*	0.79	-0.01	0.003	3.00E-05	11.9	1
Calcium channel blockers	set1	rs2488161	G	*CACNB2*	0.16	0.01	0.003	3.49E-05	13.9	1
Calcium channel blockers	set1	rs62251864	G	*CACNA1D*	0.23	0.01	0.003	5.04E-05	20.0	1
Calcium channel blockers	set1	rs73077175	A	*CACNA2D2*	0.33	-0.02	0.002	0.000123	48.6	1
Calcium channel blockers	set1	rs8065903	G	*CACNA1G*	0.74	-0.01	0.003	3.17E-05	12.6	1
Calcium channel blockers	set1	rs873839	A	*CACNA1G*	0.24	-0.01	0.003	2.53E-05	10.0	1
Calcium channel blockers	set2	rs2488136	G	*CACNB2*	0.71	-0.01	0.003	2.50E-04	187.9	0
Calcium channel blockers	set2	rs1888693	A	*CACNB2*	0.34	0.02	0.002	4.80E-04	352.8	0
Calcium channel blockers	set2	rs12258967	G	*CACNB2*	0.30	-0.03	0.003	7.20E-04	533.8	0
Calcium channel blockers	set2	rs714277	T	*CACNA1C*	0.28	0.01	0.003	2.20E-04	165.4	0
Calcium channel blockers	set2	rs150857355	C	*CACNB3*	0.02	0.05	0.008	1.00E-04	80.3	0
Calcium channel blockers	set2	rs3821843	A	*CACNA1D*	0.68	0.02	0.003	4.00E-04	296.6	0
Calcium channel blockers	set2	rs7340705	C	*CACNA1D*	0.32	0.01	0.003	2.90E-04	216.5	0
Centrally acting antihypertensives	set1	rs149028979	A	*NISCH*	0.05	0.02	0.006	2.80E-05	11.1	1
Centrally acting antihypertensives	set1	rs3755801	A	*NISCH*	0.16	0.01	0.003	2.95E-05	11.7	1
Centrally acting antihypertensives	set1	rs12416245	G	*ADRA2A*	0.19	0.01	0.003	3.08E-05	12.2	0

Centrally acting antihypertensives	set1	rs141104872	T	*NISCH*	0.06	0.02	0.005	3.43E-05	13.6	0
Centrally acting antihypertensives	set1	rs4389544	C	*GABRA2*	0.33	0.01	0.003	2.55E-05	10.1	0
Centrally acting antihypertensives	set1	rs62153010	A	*ADRA2B*	0.17	-0.01	0.003	3.62E-05	14.4	0
Loop diuretics	set1	rs78736765	T	*SLC12A2*	0.13	-0.01	0.003	3.58E-05	14.2	0
Loop diuretics	set1	rs889794	C	*SLC12A4*	0.80	-0.01	0.003	2.70E-05	10.7	0
PSDs and aldosterone antagonists	set1	rs12079515	T	*SCNN1D*	0.04	-0.02	0.006	3.06E-05	12.1	0
PSDs and aldosterone antagonists	set1	rs13303195	T	*SCNN1D*	0.53	0.01	0.002	2.65E-05	10.5	0
PSDs and aldosterone antagonists	set1	rs150274214	A	*SCNN1G*	0.02	0.03	0.009	2.76E-05	10.9	0
PSDs and aldosterone antagonists	set1	rs8048821	T	*SCNN1G*	0.50	-0.01	0.002	2.61E-05	10.3	0
Renin inhibitors	set1	rs3795573	C	*REN*	0.14	-0.01	0.003	2.80E-05	11.1	0
Thiazides and related diuretics	set1	rs4389544	C	*GABRA2*	0.33	0.01	0.003	2.55E-05	10.1	0
Thiazides and related diuretics	set1	rs140443467	G	*GABRG3*	0.03	-0.02	0.007	2.54E-05	10.1	0
Thiazides and related diuretics	set1	rs191430682	G	*GABRD*	0.04	-0.03	0.006	8.58E-05	34.0	0
Thiazides and related diuretics	set1	rs2076327	T	*GABRD*	0.49	0.01	0.002	8.80E-05	34.9	0
Thiazides and related diuretics	set1	rs4541476	T	*GABRB1*	0.41	-0.01	0.002	2.60E-05	10.3	0
Thiazides and related diuretics	set1	rs116733754	A	*GABRD*	0.01	-0.04	0.011	3.05E-05	12.1	1
Vasodilator antihypertensives	set1	rs10766393	A	*KCNJ11*	0.30	-0.01	0.003	8.11E-05	32.2	1
Vasodilator antihypertensives	set1	rs10766395	C	*KCNJ11*	0.39	-0.01	0.002	6.14E-05	24.4	1
Vasodilator antihypertensives	set1	rs11024048	C	*KCNJ11*	0.27	-0.01	0.003	3.41E-05	13.5	1
Vasodilator antihypertensives	set1	rs731152	A	*AOC3*	0.23	-0.01	0.003	3.06E-05	12.1	1
Vasodilator antihypertensives	set1	rs74835612	A	*AOC3*	0.06	0.01	0.005	2.56E-05	10.1	1
Vasodilator antihypertensives	set1	rs10305838	C	*EDNRA*	0.14	0.01	0.003	3.92E-05	15.6	0
Vasodilator antihypertensives	set1	rs11023983	C	*KCNJ11*	0.47	-0.01	0.002	3.68E-05	14.6	0
Vasodilator antihypertensives	set1	rs138643143	A	*AOC3*	0.07	0.02	0.005	5.09E-05	20.2	0
Vasodilator antihypertensives	set1	rs148451026	C	*KCNJ11*	0.03	-0.03	0.008	3.74E-05	14.8	0
Vasodilator antihypertensives	set1	rs2074311	G	*KCNJ11*	0.59	-0.01	0.002	8.41E-05	33.4	0
Vasodilator antihypertensives	set1	rs4845568	C	*NPR1*	0.95	0.02	0.005	2.82E-05	11.2	0
Vasodilator antihypertensives	set1	rs62111755	G	*PTGIR*	0.15	0.01	0.003	2.68E-05	10.6	0
Vasodilator antihypertensives	set1	rs74754758	C	*AOC3*	0.02	-0.03	0.009	3.45E-05	13.7	0

ACE, Angiotensin-converting enzyme; PSDs, potassium-sparing diuretics. *Set 1 refers to genetic variants based on the study of Walker et al.[11] in UK Biobank. In the column "Correlated", 1 refers to those with $r^2<0.8$ and were included in sensitivity analysis. As previously [16], the r^2 and F statistic was calculated using the formula in previous study [16]. Set 2 refers to genetic variants based on the study of Gill et al. [12] and Georgakis and Gill et al. [16] in meta-analysis of UK Biobank and ICBP, where the F-statistics are as reported in the referenced study.

Table S3. Associations of genetic proxies for antihypertensive drugs with eGFR in meta-analysis of UK Biobank and CKDGen incorporating correlated genetic variants

Class	#SNPs	beta	95% CI	*p*
ACE inhibitors	2	0.06	0.003, 0.11	0.04
CCBs	23	-0.01	-0.02, 0.01	0.34
Alpha-adrenoceptor blockers	7	-0.003	-0.02, 0.02	0.82
Beta-adrenoceptor blockers	14	-0.02	-0.03, -0.002	0.02
Centrally acting antihypertensives	6	0.02	-0.05, 0.10	0.53
Thiazides and related diuretics	6	-0.04	-0.08, 0.001	0.06
Vasodilator antihypertensives	13	-0.01	-0.03, 0.004	0.14

ACE, angiotensin-converting enzyme; CCB, calcium channel blocker; eGFR, estimated glomerular filtration rate; beta is the beta-coefficient with eGFR per effect size (standard deviation) of systolic blood pressure.

Table S4. Associations of genetic proxies for antihypertensive drugs with UACR in CKDGen incorporating correlated genetic variants

Class	#SNPs	beta	95% CI	*p*
ACE inhibitors	2	0.09	-0.35, 0.52	0.70
CCBs	23	-0.15	-0.28, -0.01	0.03
Alpha-adrenoceptor blockers	7	-0.10	-0.24, 0.04	0.16
Beta-adrenoceptor blockers	14	-0.04	-0.15, 0.07	0.52
Centrally acting antihypertensives	6	-0.26	-0.50, -0.03	0.03
Thiazides and related diuretics	6	-0.11	-0.48, 0.26	0.56
Vasodilator antihypertensives	13	-0.17	-0.41, 0.07	0.17

ACE, angiotensin-converting enzyme; CCB, calcium channel blocker; UACR, urine albumin-to-creatinine ratio

Table S5. Associations of genetic proxies for antihypertensive drugs with albuminuria incorporating correlated genetic variants

Class	#SNPs	Odds ratio	95% CI	*p*
CCBs	22	0.63	0.41, 0.98	0.04
Alpha-adrenoceptor blockers	7	0.77	0.42, 1.44	0.42
Beta-adrenoceptor blockers	13	0.85	0.51, 1.42	0.53
Centrally acting antihypertensives	6	0.53	0.24, 1.17	0.12
Thiazides and related diuretics	6	0.76	0.32, 1.81	0.53
Vasodilator antihypertensives	13	0.96	0.52, 1.76	0.89

CCB, calcium channel blocker; PSD, potassium-sparing diuretic

Table S6. Genetic predictors with potential pleiotropy

SNP	Relevant trait	Source (consortium/pubmed id)	Proxy for
rs34484573	Body fat percentage	UKBB	Calcium channel blockers
rs34484573	Body mass index	UKBB	
rs34484573	Qualifications: A levels or as levels or equivalent	UKBB	
rs34484573	Qualifications: college or university degree	UKBB	
rs34484573	Time spent watching television	UKBB	
rs34484573	Weight	UKBB	
rs34484573	Years of educational attainment	27225129	
rs62111755	Body mass index	28892062	Vasodilator antihypertensives
rs73077175	Body mass index	UKBB	Calcium channel blockers
rs73077175	Qualifications: A levels or as levels or equivalent	UKBB	
rs73077175	Waist circumference	UKBB	
rs73077175	Number of treatments or medications taken	UKBB	
rs873839	Serum creatinine	UKBB	Calcium channel blockers
rs9890200	Serum creatinine	UKBB	Calcium channel blockers

The latter two (rs873839 and rs9890200) were identified in summary statistics of serum creatinine in UK Biobank, the rest were identified in Phenoscanner.

Table S7. Sensitivity analysis for the association with eGFR excluding potentially pleiotropic genetic predictors*

Class	#SNPs	Meta-analysis of UK Biobank and CKDGen			UK Biobank			CKDGen		
		beta	95% CI	*p*	beta	95% CI	*p*	beta	95% CI	*p*
Calcium channel blockers	7	-0.004	-0.02, 0.01	0.65	-0.01	-0.03, 0.02	0.56	-0.001	-0.02, 0.02	0.97
	19	-0.006	-0.02, 0.01	0.41	-0.006	-0.02, 0.01	0.51	-0.007	-0.03, 0.02	0.58
Vasodilator antihypertensives	7	-0.01	-0.04, 0.01	0.33	-0.02	-0.05, 0.02	0.28	-0.005	-0.04, 0.03	0.80

*These genetic predictors excluded were shown in Supplemental Table 6. For calcium channel blockers, rs34484573 and rs9890200 were excluded when using uncorrelated SNPs, rs34484573, rs73077175, rs873839, and rs9890200 were excluded when incorporating correlated SNPs

Table S8. Sensitivity analysis for the association with UACR and albuminuria excluding potentially pleiotropic genetic predictors*

Class	UACR				Risk of albuminuria			
	#SNPs	beta	95% CI	*p*	#SNPs	Odds ratio	95% CI	*p*
Calcium channel blockers	8	-0.18	-0.33, -0.04	0.01	8	0.53	0.32, 0.89	0.02
	21	-0.20	-0.34, -0.06	0.01	20	0.53	0.35, 0.81	0.003
Vasodilator antihypertensives	7	-0.05	-0.23, 0.12	0.55	7	1.28	0.69, 2.37	0.44

*These genetic predictors excluded were shown in Supplemental Table 6. For calcium channel blockers, rs34484573 was excluded when using uncorrelated SNPs, rs34484573 and rs73077175 were excluded when incorporating correlated SNPs

Table S9. Sensitivity analysis on the associations of genetic proxies for antihypertensive drugs with eGFR using published genetic variants derived from UK Biobank in Mendelian randomization using different analysis methods

Class	Methods	#SNPs	Meta-analysis of UK Biobank and CKDGen			UK Biobank			CKDGen		
			beta	95% CI	*p*	beta	95% CI	*p*	beta	95% CI	*p*
CCBs	WM	9	-0.007	-0.03, 0.01	0.48	-0.01	-0.04, 0.02	0.43	-0.003	-0.03, 0.02	0.81
	MR PRESSO		-0.004	-0.02, 0.01	0.57	-0.006	-0.03, 0.01	0.51	-0.001	-0.02, 0.02	0.94
Alpha-adrenoceptor blockers	WM	6	-0.01	-0.04, 0.01	0.26	-0.02	-0.05, 0.01	0.30	-0.01	-0.04, 0.02	0.59
	MR PRESSO		-0.004	-0.03, 0.02	0.73	-0.01	-0.06, 0.04	0.56	0.002	-0.04, 0.05	0.91
Adrenergic neuron blockers	WM	3	-0.03	-0.09, 0.04	0.41	-0.06	-0.12, 0.01	0.09	0.01	-0.06, 0.08	0.85
Beta-adrenoceptor blockers	WM	7	-0.03	-0.05, -0.01	0.01	-0.03	-0.06, -0.003	0.03	-0.02	-0.05, 0.005	0.10
	MR PRESSO		-0.02	-0.04, -0.004	0.02	-0.04	-0.08, 0.005	0.07	-0.02	-0.03, 0.001	0.06
Centrally acting antihypertensives	WM	4	-0.01	-0.09, 0.06	0.73	-0.05	-0.10, 0.004	0.07	0.03	-0.03, 0.09	0.40
	MR PRESSO		-0.01	-0.09, 0.07	0.82	-0.04	-0.08, -0.01	0.02	0.04	-0.07, 0.14	0.33
PSDs and aldosterone antagonists	WM	4	-0.03	-0.08, 0.02	0.22	-0.04	-0.10, 0.02	0.22	-0.02	-0.08, 0.05	0.64
	MR PRESSO		-0.03	-0.06, -0.001	0.05	-0.04	-0.12, 0.05	0.27	-0.03	-0.09, 0.03	0.24
Thiazides and related diuretics	WM	4	-0.03	-0.09, 0.04	0.40	-0.06	-0.11, -0.01	0.02	0.01	-0.05, 0.06	0.82
	MR PRESSO		-0.01	-0.07, 0.05	0.79	-0.04	-0.13, 0.04	0.20	0.02	-0.01, 0.05	0.31
Vasodilator antihypertensives	WM	8	0.002	-0.03, 0.03	0.90	-0.01	-0.05, 0.03	0.69	0.01	-0.03, 0.05	0.56
	MR PRESSO		-0.01	-0.03, 0.02	0.57	-0.01	-0.05, 0.03	0.55	-0.003	-0.05, 0.04	0.86

CCB, calcium channel blocker; WM, weighted median

Table S10. Sensitivity analysis on the associations of genetic proxies for antihypertensive drugs with albuminuria and UACR using genetic variants derived from UK Biobank in Mendelian randomization using different analysis methods

Class	Methods	Albuminuria				UACR			
		#SNPs	OR	95% CI	P value	#SNPs	beta	95% CI	P value
CCBs	WM	8	0.54	0.32, 0.91	0.02	9	-0.21	-0.37, -0.06	0.01
	MR PRESSO		0.58	0.34, 0.97	0.046		-0.15	-0.30, 0.007	0.06
Alpha-adrenoceptor blockers	WM	6	0.63	0.33, 1.22	0.17	6	-0.18	-0.37, 0.02	0.08
	MR PRESSO		0.81	0.33, 1.99	0.57		-0.10	-0.30, 0.09	0.23
Adrenergic neuron blockers	WM	3	1.34	0.37, 4.76	0.66	3	-0.18	-0.57, 0.20	0.35
Beta-adrenoceptor blockers	WM	7	0.59	0.32, 1.11	0.10	7	-0.03	-0.20, 0.15	0.77
	MR PRESSO		0.52	0.27, 1.01	0.06		-0.04	-0.19, 0.12	0.57
Centrally acting antihypertensives	WM	4	0.63	0.20, 1.92	0.41	4	-0.25	-0.56, 0.06	0.12
	MR PRESSO		0.54	0.12, 2.49	0.29		-0.24	-0.32, -0.17	0.002
PSDs and aldosterone antagonists	WM	3	1.31	0.29, 5.83	0.72	4	-0.07	-0.46, 0.32	0.73
	MR PRESSO						-0.14	-0.82, 0.55	0.57
Thiazides and related diuretics	WM	4	0.77	0.30, 1.95	0.58	4	-0.05	-0.36, 0.26	0.74
	MR PRESSO		0.81	0.17, 3.86	0.69		-0.35	-0.84, 0.14	0.15
Vasodilator antihypertensives	WM	8	1.49	0.69, 3.19	0.31	8	-0.004	-0.23, 0.22	0.97
	MR PRESSO		1.04	0.44, 2.48	0.92		-0.07	-0.26, 0.12	0.41

CCB, calcium channel blocker; WM, weighted median

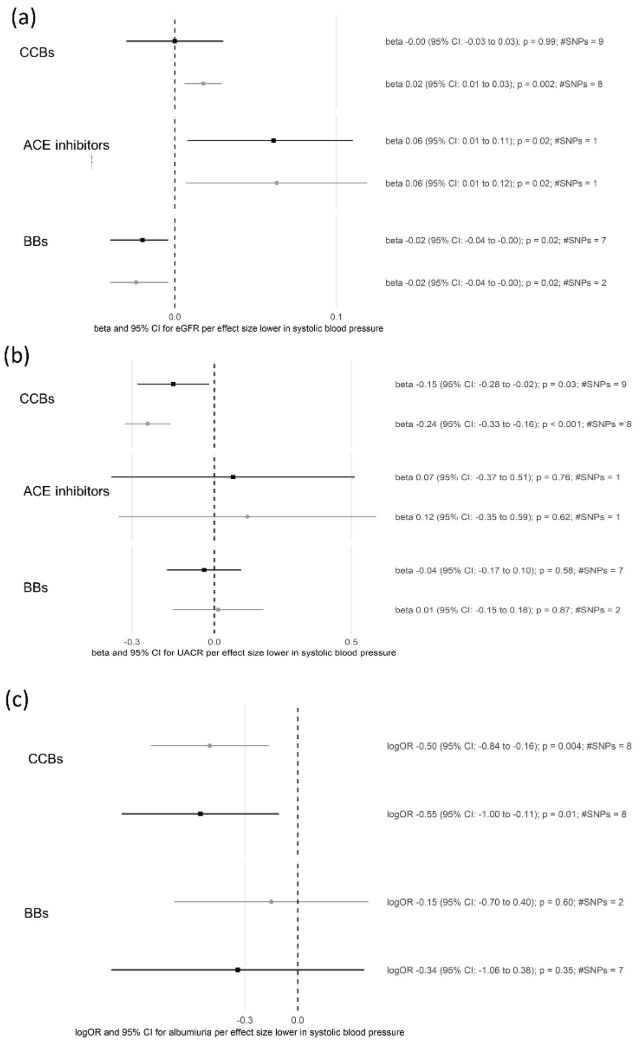

Figure S1. Comparison using different sets of genetic proxies for ACE inhibitors, CCBs and BBs. (a) for eGFR, (b) for UACR, (c) for albuminuria. Black square refers to using genetic variants based on the study of Walker et al. [11] in UK Biobank, grey circle refers to using genetic variants based on the study of Gill et al. [12] and Georgakis and Gill et al. [16] in meta-analysis of UK Biobank and ICBP.

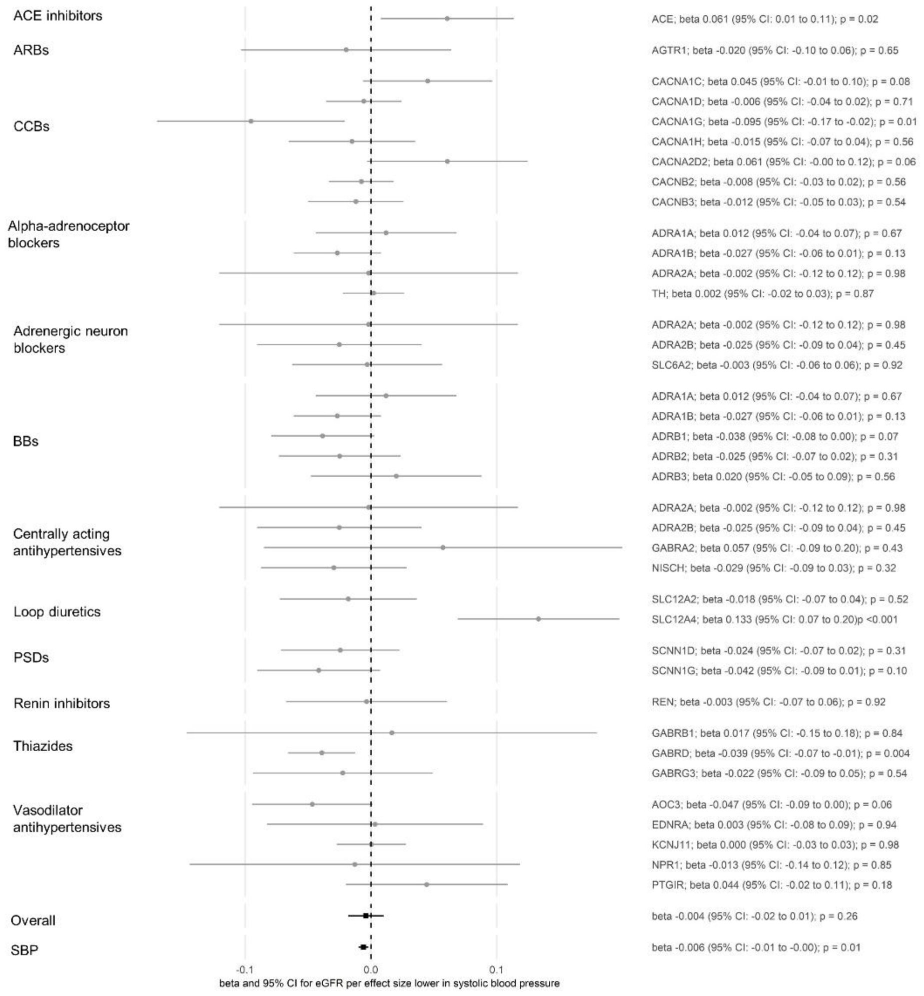

Figure S2. Associations of genetic proxies for antihypertensive drugs with eGFR by drug target in each drug class, overall antihypertensives and systolic blood pressure. SBP, systolic blood pressure. ACE, Angiotensin-converting enzyme; ARB, Angiotensin II Receptor Blocker; BBs, beta-adrenoceptor blockers; CCBs, calcium channel blockers; PSDs, potassium-sparing diuretics; SBP, systolic blood pressure.

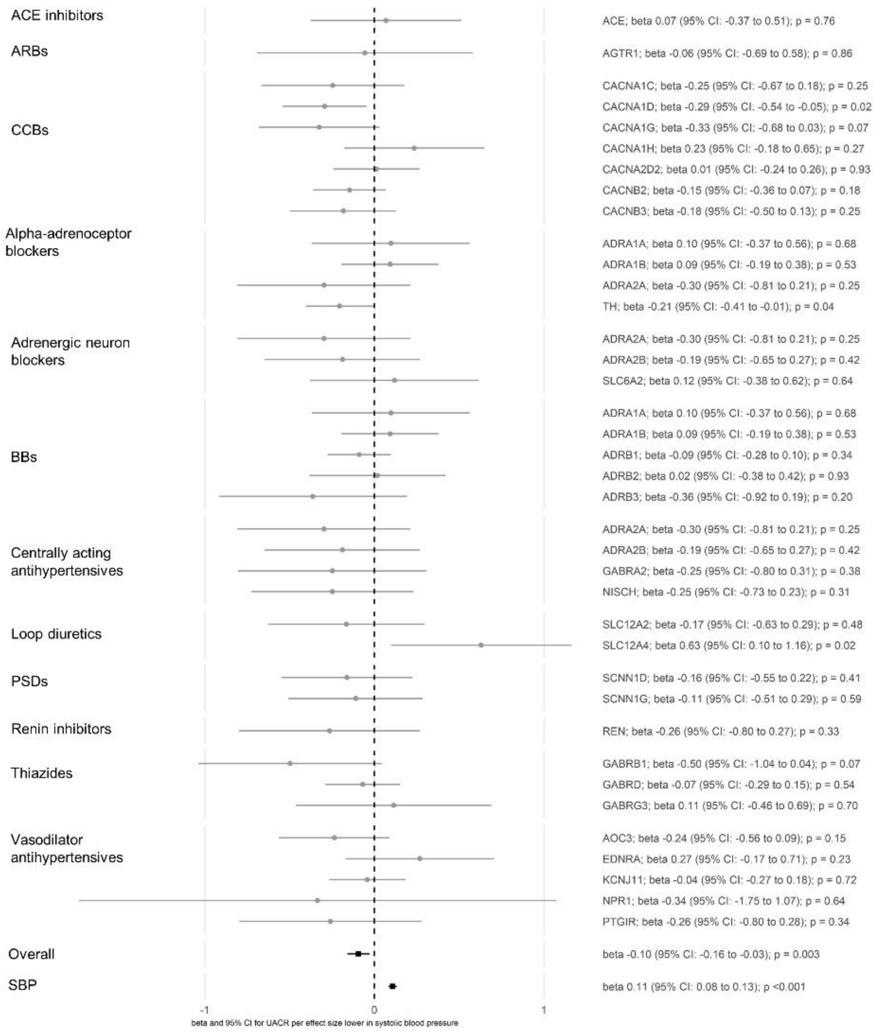

Figure S3. Associations of genetic proxies for antihypertensive drugs with UACR by drug target in each drug class, overall antihypertensives and systolic blood pressure. ACE, Angiotensin-converting enzyme; ARB, Angiotensin II Receptor Blocker; BBs, beta-adrenoceptor blockers; CCBs, calcium channel blockers; PSDs, potassium-sparing diuretics; SBP, systolic blood pressure

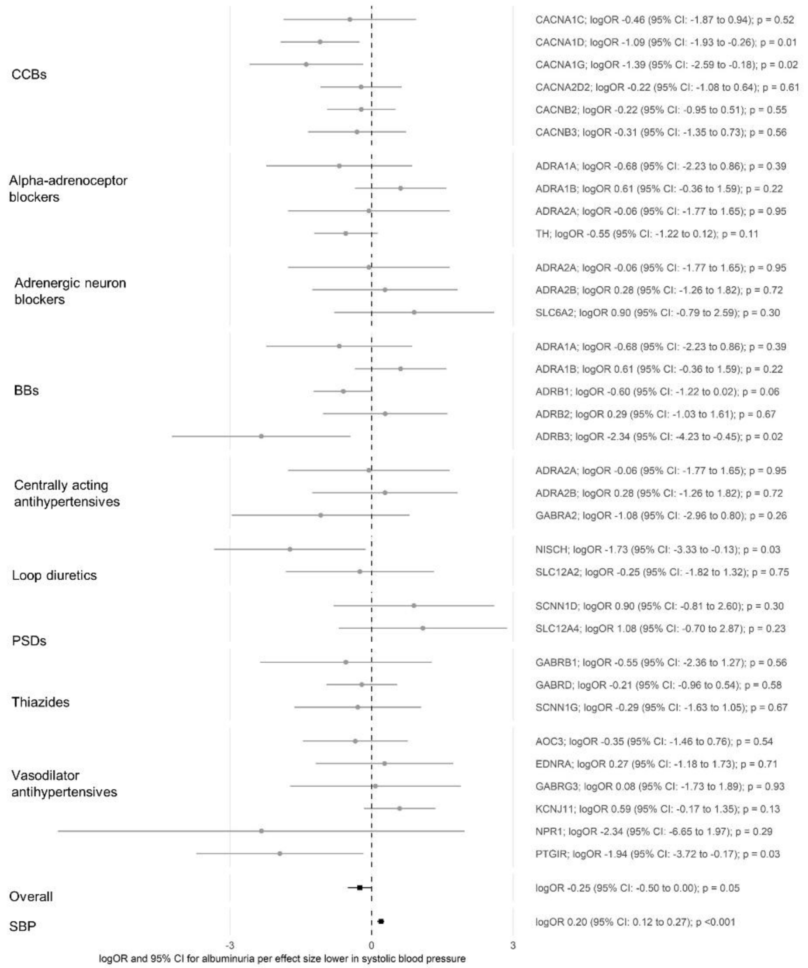

Figure S4. Associations of genetic proxies for antihypertensive drugs with albuminuria by drug target in each drug class, overall antihypertensives and systolic blood pressure. BBs, beta-adrenoceptor blockers; CCBs, calcium channel blockers; PSDs, potassium-sparing diuretics; SBP, systolic blood pressure.
